# The complete chloroplast genome of Ilex ‘Emily Bruner’, Ilex cornuta ‘Burfordii’ × Ilex latifolia (Aquifoliaceae)

**DOI:** 10.1080/23802359.2020.1810146

**Published:** 2020-08-26

**Authors:** Fan Zhang, Yunlong Li, Linhe Sun, Yanwei Zhou, Hong Chen, Xiaoqing Lu, Naiwei Li, Chuanyong Wang

**Affiliations:** Jiangsu Key Laboratory for the Research and Utilization of Plant Resources, Institute of Botany, Jiangsu Province and Chinese Academy of Sciences, Nanjing Botanical Garden Mem. Sun Yat-Sen, Nanjing, China

**Keywords:** Ilex ‘Emily Bruner’, complete chloroplast genome phylogenetic analysis

## Abstract

*Ilex* ‘Emily Bruner’ is an important economic plant with ornamental and ecological functions in southeastern China. In this study, we characterized the complete chloroplast (cp) genome sequence of ‘Emily Bruner’ to investigate its phylogenetic relationship. The entire cp genome of ‘Emily Bruner’ was 157,216 bp in length with 37.68% overall GC content, including a large single-copy (LSC) region of 86,721 bp and a small single-copy (SSC) region of 18,427 bp, which were separated by a pair of inverted repeats (IRs) of 52,068 bp. The cp genome contained 135 genes, including 90 protein-coding genes, 37 tRNA genes, and 8 rRNA genes. Phylogenetic analysis based on whole cp genome sequences showed that ‘Emily Bruner’ is closest to *I. cornuta* species.

*Ilex* ‘Emily Bruner’, also known as ‘Emily Bruner’ holly, is an artificial hybrid between *I. cornuta* Lindl. and Paxt. ‘Burfordii’ and *I. latifolia* Thunb., which has been widely spread in America, Europe and Asia (Briggs et al. 2002). It was introduced to southeastern China in 1990s for ecological, economical and ornamental purposes. However, due to the similar leave and fruit with other species and cultivars, it is difficult to be distinguished and identified by morphology (Yao et al. 2016). As an effective DNA molecular marker, the chloroplast genome has been widely used in genetic and evolutionary relationships studies in plants (Freitas et al. 2018). In this study, we reported the complete chloroplast genome sequence of ‘Emily Bruner’ with bioinformatics analysis, which would be helpful for further research on the identification and classification of genus *Ilex*.

Samples of ‘Emily Bruner’ were collected from Nanjing Botanical Garden, Mem. Sun Yat-sen (118°49′55″E, 32°3′32″N), Nanjing, China. The voucher specimen (NO. NBGJIB-Ilex-0018) was deposited in the Institute of Botany, Jiangsu Province and Chinese Academy of Science. Genomic DNA was extracted using the GMS16011.2.1 Kit (Genmed Scientifics Inc., USA). A paired-end library with an insert-size of 350-bp was constructed and sequenced on the Illumina NovaSeq system (Illumina, San Diego, CA). In total, 5006.4 Mb of raw data (4778.9 Mb clean data) were obtained. *De novo* genome assembly and annotation were conducted by NOVOPlasty (Dierckxsens et al. 2017) and GeSeq (Tillich et al. 2017), respectively. The annotated cp genome was deposited in GenBank (accession number: MT417234).

The whole cp genome sequence of ‘Emily Bruner’ was 157,216 bp in length, containing a large single-copy region (LSC) region of 86,721 bp, a small single-copy region (SSC) of 18,427 bp, and a pair of inverted repeats (IRs, including IRa and IRb) of 52,068 bp. The ‘Emily Bruner’ cp genome contained 135 genes, including 39 transfer RNA genes, 8 ribosomal RNA genes and 90 protein-coding genes. Among them, 12 splitting genes contained introns and exons and two of them (ycf3, clpP) had two introns, others had a single intron. The overall GC content of the cp genome was 37.64%.

In a previous study, 10 cp genomes of *Ilex* were completed, which including the parents of ‘Emily Bruner’(Yao et al. 2016; Cascales et al. 2017; Park et al. 2019). To reveal the phylogenetic position of ‘Emily Bruner’ with these members, a phylogenetic analysis was performed based on 10 complete cp genomes, and one taxa from *Helwingia* was served as outgroup. The neighbor-joining (bootstrap repeat is 10,000) and maximum likelihood (bootstrap repeat is 1000) were used for constructing phylogenetic trees using PhyML v3.0 (http://www.atgc-montpellier.fr/phyml/) (Liu et al. 2019). The phylogenetic tree showed that ‘Emily Bruner’ closely related to its female parent *I. cornuta* species (Figure 1). The cp genome sequence of ‘Emily Bruner’ in this study will be useful for further analysis on molecular markers and molecular breeding.

**Figure 1.**
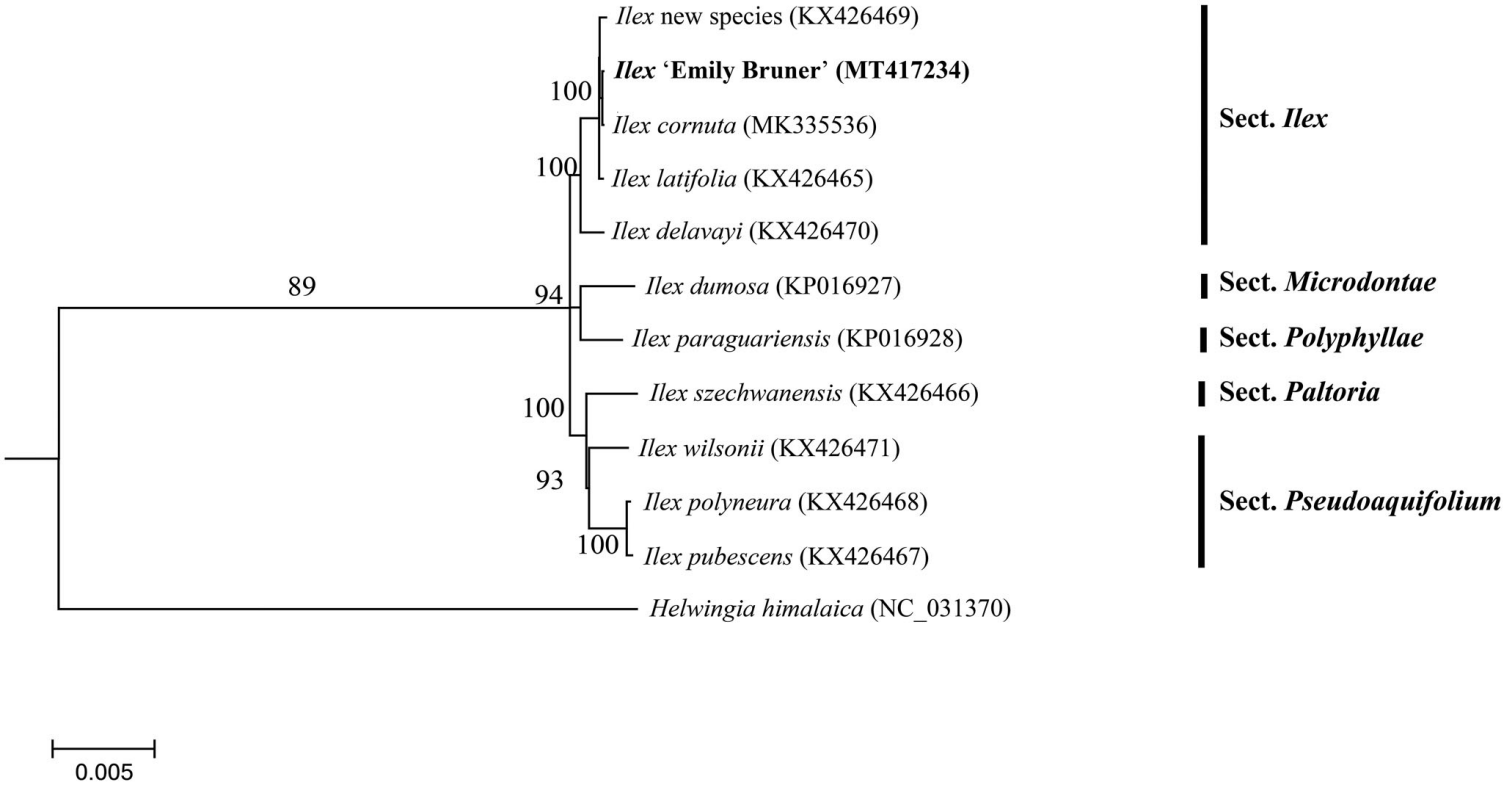
Maximum likelihood phylogenetic tree based on the sequences of 11 complete chloroplast genomes, showing the Ilex ‘Emily Bruner’ is closer with I. cornuta species. Section names were displayed in the right side of phylogenetic tree (Gottlieb et al. 2005; Jiang et al. 2017).

## Data Availability

The data that support the findings of this study are openly available in NCBI at http://www.ncbi.nlm.nih.gov/, reference number MT417234.
